# Sudden cardiac arrest during Nuss procedure for pectus excavatum

**DOI:** 10.1186/s13019-020-01180-5

**Published:** 2020-06-12

**Authors:** Do Yeon Kim, Jin Yong Jeong

**Affiliations:** Department of Thoracic and Cardiovascular Surgery, Incheon St. Mary’s Hospital, College of Medicine, The Catholic University of Korea, 56 Dongsu-ro, Bupyeong-gu, Incheon, 21431 Republic of Korea

**Keywords:** Pectus excavatum, Nuss procedure, Complications, Cardiac arrest

## Abstract

Cardiac arrest during the Nuss procedure is the most serious complication and is related to cardiac injury by the surgical instruments and pectus bars. To avoid the cardiac injury, there are several techniques with various devices, including crane and wire suture, lifting hook, the Kent or Langenbeck retractor, and the Vacuum Bell device. However, a case of cardiac arrest without direct cardiac injury during the Nuss procedure has been reported in the pectus excavatum patient with coronary-to-pulmonary arterial shunts. Recently, we encountered a case of cardiac arrest without cardiac abnormalities in preoperative studies and cardiac injury during the Nuss procedure.

**Correspondence.**

**Dear Sir,**

We read with great interest the report by Zou et al. presenting the case of cardiac arrest without physical cardiac injury during the Nuss procedure [1]. A life-threatening complication during the Nuss procedure is cardiac injury by the surgical instruments and pectus bars, which may result in cardiac arrest [2, 3]. Zou et al. encountered cardiac arrest twice during a modified Nuss procedure, when pulling the pectus bar and after rotating the bar, in the patient with pectus excavatum. The patient’s preoperative echocardiography showed two abnormal transverse shunts classified as coronary-to-pulmonary arterial shunts. Recently, we also encountered a case of sudden cardiac arrest without cardiac injury during the Nuss procedure in the pectus excavatum patient, whose preoperative electrocardiogram and echocardiogram showed no abnormality.

A 17-year-old male presented with depressed chest deformity since childhood without special medical history. Chest computed tomography scans revealed symmetrical depression of the lower anterior chest wall with indentation on the heart and a Haller index of 4.0 (Fig. 1a). Preoperative electrocardiography showed sinus rhythm with right atrium enlargement and borderline right axis deviation. Preoperative echocardiography demonstrated normal cardiac chamber dimensions and normal left and right ventricular contractility. The patient underwent the Nuss procedure in supine position. When dissecting the substernal space after applying the crane technique to lift the sternum to avoid cardiac injury [4], ventricular fibrillation occurred suddenly after the appearance of bradycardia and premature ventricular contractions (Fig. 1b). Sinus rhythm was restored after immediate cardiopulmonary resuscitation including chest compressions and administrations of an electric cardioversion and anti-arrhythmic agents (Fig. 1c). The operation was terminated and the patient was transferred to the intensive care unit. A cardiologist recommended further evaluations but the patient refused the recommendation and was discharged with good recovery 4 days later. The patient visited our hospital for 3 years without surgical complications.

**Fig. 1:**
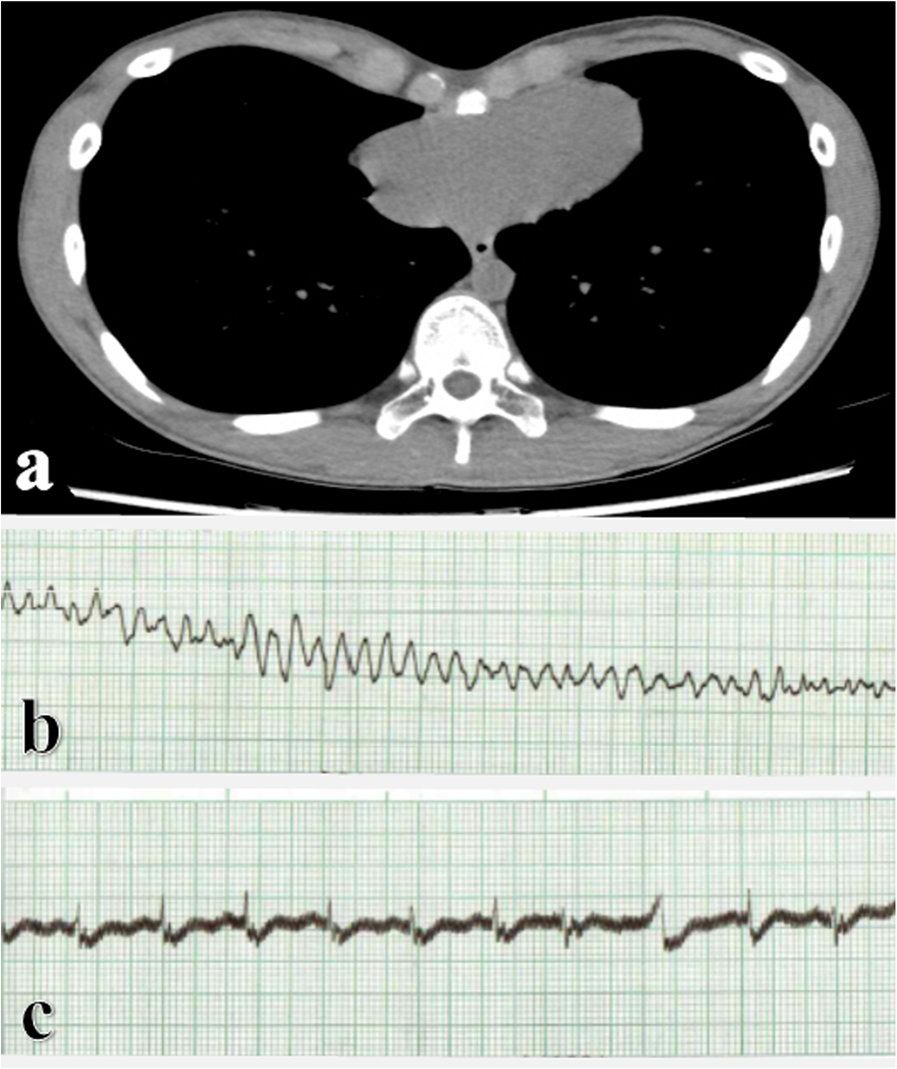
**a** The preoperative chest computed tomography scans showing symmetrical depression of the anterior chest wall and cardiac indentation. **b** Electrocardiographic monitoring in the operating room revealing ventricular fibrillation during the Nuss procedure and **c** Sinus rhythm restoring after immediate cardiopulmonary resuscitation

Cardiac arrest during the Nuss procedure is mostly related to cardiac injury such as cardiac perforation and physical stimulation to the heart with a surgical instrument and oppression of the right ventricular outflow. Several techniques have been used to avoid cardiac injury during the Nuss procedure [4–6]. Lifting the sternum is the key to releasing the substernal space in which the pectus bar will be inserted and placed. Various devices, including crane and wire suture, lifting hook, the Kent or Langenbeck retractor, and the Vacuum Bell device, have been used to lift the sternum.

Cardiac arrest not related to cardiac injury during the Nuss procedure rarely occurs. Zou et al. hypothesized two possible mechanisms in their case [1]. First, rotation of the heart by sternal elevation twisted the coronary-to-pulmonary arterial shunts, resulting in directional change of blood flow in the shunts, caused acute myocardial ischemia and consequent ventricular fibrillation. Second, nerve stretching caused by sudden enlargement of the substernal space may upset the balance between vagal and sympathetic innervation, which triggers inhibition of cardiac function and consequent arrest. In our case, preoperative eletrocardiography and echocardiography revealed no abnormal findings and we thought that cardiac arrest of our case may be related to nerve stretching caused by sternal elevation.

In summary, we encountered a case of cardiac arrest without cardiac injury during the Nuss procedure in the pectus excavatum patient with normal electrocardiogram and echocardiogram. We would like to emphasize that cardiac arrest may occur due to sternal elevation even if there is no abnormality in the preoperative cardiac examination and no cardiac injury during the Nuss procedure.

## Data Availability

Not applicable.
